# The role of mammalian target of rapamycin pathway in the pathogenesis of pauci-immune glomerulonephritis

**DOI:** 10.1080/0886022X.2019.1667829

**Published:** 2019-10-28

**Authors:** Zeki Soypacaci, Ozlem Cakmak, Fulya Cakalagoglu, Onay Gercik, Ibrahim Ertekin, Atilla Uzum, Rifki Ersoy, Servet Akar

**Affiliations:** a Department of Nephrology, Izmir Katip Celebi University, Izmir, Turkey;; b Department of Internal Medicine, Izmir Katip Celebi University, Izmir, Turkey;; c Department of Pathology, Izmir Katip Celebi University, Izmir, Turkey;; d Department of Rheumatology, Izmir Katip Celebi University, Izmir, Turkey

**Keywords:** Pauci-immune glomerulonephritis, mTOR protein, PTEN protein, latency associated protein TGF B1, pathology

## Abstract

**Background:** The characteristic lesion of pauci-immune glomerulonephritis is focal necrotizing and crescentic glomerulonephritis. The underlying mechanisms in the formation or progression of crescent formation need further investigations. Therefore, we aimed to evaluate the role of mammalian target of rapamycin (mTOR), which might be a potential therapeutic target, in kidney biopsies of patients with pauci-immune glomerulonephritis.

**Methods:** The patients diagnosed as pauci-immune glomerulonephritis at an outpatient nephrology clinic were retrospectively reviewed and those patients who had a kidney biopsy before receiving an immunosuppressive treatment were included in the study. Kidney biopsy specimens were immunohistochemically stained with mTOR, antibodies of phosphatase and tensin homolog (PTEN) and transforming growth factor-β (TGF-β) and scored by an experienced renal pathologist.

**Results:** In total, 54 patients with pauci-immune glomerulonephritis (28 [52%] female) were included. According to the histopathologic examination, 22% of our cases were classified as focal, 33% crescentic, 22% mixed, and 22% as sclerotic. The mTOR was expressed in substantial percentages of glomeruli of patients with pauci-immune glomerulonephritis. However, we observed PTEN expression in all samples and mTOR in all tubulointerstitial areas. mTOR expression was found to be related with the presence of crescentic and sclerotic changes observed in glomeruli and the degree of fibrosis in interstitial areas. Serum creatinine level or response to treatment was not found to be associated with mTOR pathway expression.

**Conclusion:** Our results suggest that mTOR pathway may play role in the pathogenesis of pauci-immune glomerulonephritis, besides targeting this signaling may be an alternative option for those patients.

## Introduction

Pauci-immune glomerulonephritis is the most common form of rapidly progressive glomerulonephritis in adults. The characteristic lesion of the disease is focal necrotizing and crescentic glomerulonephritis. It may be a component of systemic small vessel vasculitis or kidney-limited disease [1]. If it is not treated appropriately; cellular crescents rapidly turn into fibrous crescents, glomerular capillary collapse occurs, and end-stage renal failure develops [2].

Mammalian target of rapamycin (mTOR) is a serine/threonine kinase and plays role in the regulation of cell growth and proliferation. It also regulates cell survival and is stimulated by growth factors, nutrients, stress signals, phosphatidylinositol-4,5-bisphosphate 3-kinase(PI3K), mitogen-activated protein kinase(MAPK), 5' adenosine monophosphate(AMP), and 5' adenosine monophosphate-activated protein kinase (AMPK). mTOR complex includes two multiprotein complexes; mTOR complex 1 (mTORC1) and mTOR complex 2 (mTORC2) [3]. mTORC1 activates a number of substrates like ribosomal subunit-6 kinase-1 (S6K1) and eucaryotic initiation factor 4E (eIF4E) binding protein-1 (4EBP1), which are responsible for mRNA translation [4]. mTORC2 regulates actin cytoskeleton and activates protein kinase C-α (PKC-α) and AKT (protein kinase B; PKB). mTOR multiprotein complexes have a positive effect on fibrotic interleukins (IL). Liang et al. [3] showed that IL-4, IL-6, IL-17, and TGF-β were decreased after mTOR inhibition with rapamycin. Phosphatase and tensin homolog (PTEN) is the negative regulator of the AKT/mTOR pathway [5]. It usually inhibits mTOR by inhibiting AKT. Decreased intracellular levels of PTEN cause PI3K/AKT/mTOR pathway activation and increased cell proliferation, survival, adhesion, migration, and angiogenesis [6].

The stimulation of the TGF-β receptor activates intracellular pathways via both PI3K and suppressor of mothers against decapentaplegic (SMAD). With the TGF-β receptor, the phosphorylation of the PI3K molecule activates AKT, mTOR, and S6K1 [7,8]. This continues with S6 kinase activation and translation enhancement [7]. The result is an increase in protein synthesis and cell migration.

In systemic autoimmune diseases, several molecular signaling pathways such as MAPK, AKT, and nuclear factor kappa B have been shown to be active. Within all these molecular pathways, PI3K/AKT/mTOR pathway emerges due to its being a therapeutic target [9]. Previously some evidences have been obtained regarding dysregulation of mTOR signaling in some prevalent kidney diseases like diabetic nephropathy and cystic kidney disease and the association of increased fibrosis with mTOR in chronic kidney disease [10–15]. However, previously the role of mTOR pathway in pauci-immune glomerulonephritis pathogenesis was evaluated in only one study. To this end, we aimed to determine the role of mTOR pathway in the pathologic changes observed in kidney biopsies of patients with pauci-immune glomerulonephritis.

## Materials and methods

### Patients and data collection

The patients who admitted to outpatient nephrology clinic of a tertiary hospital with a diagnosis of pauci-immune glomerulonephritis between May 2009 and June 2016 were retrospectively reviewed by using hospital records. Those patients who had undergone renal biopsy before receiving any immunosuppressive treatment including mTOR inhibitors and aged ≥18 years were included in the study. Baseline data including demographic, clinical, and serum auto-antibody results were obtained. Treatment(s) applied, pre- and post-treatment (12th month) serum creatinine values were collected from patients charts. Treatment response was defined as (a) stabilization or improvement in serum creatinine after treatment and (b) not requirement of renal replacement therapy. The final survival status of the patients at the time of data collection and causes of the death were also recorded.

### Histopathological evaluation

All of the biopsy specimens had been previously examined by light microscopy and immunofluorescence. All kidney biopsies, which were fixed with 10% buffered formalin for at least 6 h, were monitored with a closed-loop tissue monitoring machine overnight. One block was selected for each patient from these paraffin-embedded blocks. Serial sections of 4–5 micron thickness obtained from these blocks were stained with Hematoxylin Eosin (H&E) and reexamined blindly by light microscope by a single expert pathologist (FC). The specimens were evaluated for the cellularity, presence of crescents, necrosis, and glomerular sclerosis and also classified as suggested Berden et al. [16]. When presenting the histologic model, Berden et al. suggested that at least 10 whole glomeruli needed to be present for an adequate classification. However, Rune Bjørneklett's study showed that there was no difference in the classification of patients with pauci-immune glomerulonephritis between 10 and over glomeruli in renal biopsies and those with between 3 and 9 glomeruli [17], so we also examined pauci-immune glomerulonephritis patients with at least 3 and over glomeruli in renal biopsies we included in the study.

### Immunohistochemical staining

Renal biopsy specimens were evaluated by immunohistochemical staining with antibodies of PTEN (Spring Bioscience Rabbit Anti-Human PTEN Monoclonal Antibody, Clone SP218), mTOR (Spring Bioscience Rabbit Anti-Human mTOR Polyclonal Antibody), and TGF-β1 (Spring Bioscience Rabbit Anti-Human Transforming Growth Factor 1 β Polyclonal Antibody) by a single expert pathologist (FC). Positive control tissues were prostate adenocarcinoma tissue for PTEN, placenta for TGF-β1, and breast cancer tissue for mTOR. Immunohistochemical staining slides were evaluated via light microscopy. Positive staining was described as brown colored staining in cytoplasm and cell membrane.

### Immunohistochemical evaluation

**Figure 1.**
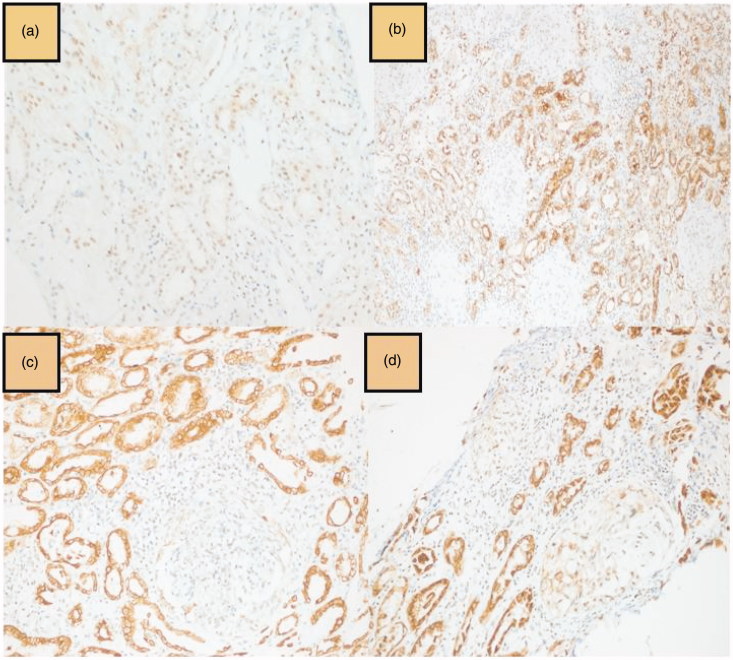
Tubulointerstitial and glomerular mTOR immunostaining; mild (a) and strong (b) tubulointerstitial mTOR positivity, and mild (c), and moderate (d) glomerular mTOR positivity.

**Figure 2.**
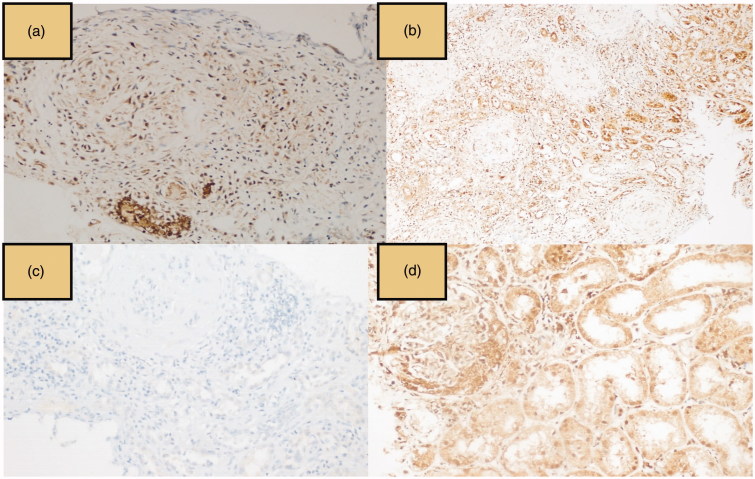
Phosphatase and tensin homolog (PTEN) and transforming growth factor-β1 (TGF-β1) immunostaining; PTEN moderate-positive staining (a) and strong-positive staining (b), TGF-β1 negative staining (c), and strong-positive staining (d).

Immunohistochemical mTOR staining was evaluated and scored separately in glomerular and tubulointerstitial area. mTOR staining intensity was semi-quantitatively graded as mild (Figure 1(a,c)), moderate, and strong (Figure 1(b,d)) positivity. The percentage of mTOR staining cells was also rated as following: 0 (<5%), 1 (5–25%), 2 (26–50%), and 3 (51–75%) [18,19]. Cellular staining of PTEN was evaluated as negative, mild, moderate and strong positive (Figure 2(b)) [19]. Immunohistochemical staining of TGF-β1 was graded as following; negative, 1–25, 26–50, 51–75, and 76–100% [20].

**Figure 3.**
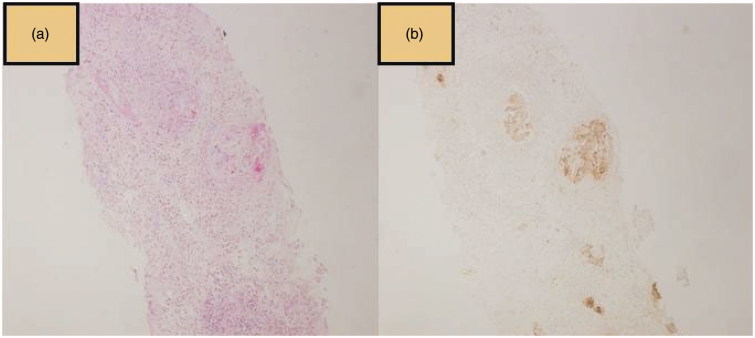
Hematoxylin eosin (a) and immunohistochemically mTOR (b) stained images of the crescentic glomerule.

### Statistical analysis

Unless otherwise stated, results are presented as mean and standard deviation (SD) or as percentages, as appropriate. Chi-square and Spearman’s rank correlation tests were performed for comparing categorical data and for bivariate correlations. Paired sample *t* test was used to compare the before and after treatment values. All tests were two-tailed and for all measurements a *p* values of <.05 was considered statistically significant. All statistical analyses were performed by using Statistical Package of Social Science (SPSS), version 16.0 software (Chicago, IL).

## Results

In total, formalin-fixed sections of kidney biopsies of 54 patients with pauci-immune glomerulonephritis (28 [52%] female) were included in the study. All of the biopsy samples had three or more glomeruli and the mean number of glomeruli was 11.8 ± 6.2 (The crescentic glomerular appearance is shown in Figure 3). Only nine patients had 3–8 glomeruli.

Some of the demographic and clinical features of the patients with pauci-immune glomerulonephritis are summarized in Table 1.

**Table 1.** Demographic and some of the clinical and laboratory features of study group.

Characteristic	(*n* = 54)
Age, year (mean ± SD)	54.4 ± 14.4
Female, %	52
Baseline serum creatinine, mg/dL (mean ± SD)	4.5 ± 2.2
First year serum creatinine, mg/dL (mean ± SD)	2.8 ± 2.2
ANCA positivity, (*n*) (%)	32 (59)
Immunsupresive treatment (*n*)	
Corticosteroid	46
Cyclophosphamide	39
Azathioprine	10
Rituximab	7
Eculizumab	2

In total, of 32 patients which were determined ANCA positive; 20 (63%) patients were cytoplasmic (c-) ANCA or proteinase-3 ANCA positive and 12 (37%) patients were perinuclear (p-) or myeloperoxidase (MPO) ANCA positive. Twenty-five (46%) patients were diagnosed as granulomatosis with polyangiitis, 6 (11%) patients as microscopic polyangiitis, 16 (30%) patients as renal-limited disease, one (2%) patient as eosinophilic granulomatosis with polyangiitis. Six (11%) patients with pauci-immune glomerulonephritis could not be classified definitely.

According to the histopathologic examination, 12 (22%) cases were classified as focal, 18 (33%) cases were classified as crescentic, 12 (22%) cases were classified as mixed, and 12 (22%) cases were classified as sclerotic. Seven patients (13%) died <1 year after being diagnosed with pauci-immune glomerulonephritis and two patients died after more than 1 year of follow-up. After the diagnosis seven patients (13%) were moved elsewhere or to the other center however all of them were still alive according the national registers of persons at the time of the preparation of manuscript to the publication. In the first year of follow-up, serum creatinine level was decreased significantly (*p* < .001) and during follow-up 12 patients were diagnosed as end-stage renal disease. Among patients who followed up more than 1 year 32/40 (80%) were found to be responsive to treatment in accordance with the description made.

PTEN and mTOR (glomerular and tubulointerstitial area) expression intensity by immunohistochemical staining were shown in Table 2. Tubulointerstitial areas of all samples were found to be positive for mTOR expression. PTEN was expressed in all biopsy samples although mildly in most of the cases. Percentages of cells stained by mTOR in both glomerular and tubulointerstitial area were shown in Table 3. Staining by mTOR was seen in a larger percentage of cells in tubulointerstitial area than glomerular cells. In 11 (20%) of kidney biopsy samples were not stained by TGF-β1. However in 56% of our samples, TGF-β1 staining was observed in <25% of cells. In 22% of samples, 26–50% of cells stained by TGF-β1 and in 1 patient >50% of cells were positive.

**Table 2.** Immunohistochemical staining intensities of mTOR (glomerular and tubulointerstitial area) and PTEN in renal biopsy samples of patients with pauci-immune glomerulonephritis.

	Negative	Mild positivity	Moderate positivity	Strong positivity
Glomerular mTOR staining intensity, %	18	74	8	–
Tubulointerstitial mTOR staining intensity, %	–	26	41	33
PTEN staining intensity, %	–	57	23	20

mTOR: mammalian target of rapamycin; PTEN: phosphatase tensin homolog.

**Table 3.** Percentages of cells stained by mTOR in glomerular and tubulointerstitial areas of renal biopsy samples of patients with pauci-immune glomerulonephritis.

	<5%	6–25%	26–50%	51–75%	>75%
Glomerular mTOR staining, %	29	49	14	6	2
Tubulointerstitial mTOR staining, %	2	18	38	38	4

mTOR: mammalian target of rapamycin.

### Correlation analysis

Glomerular mTOR expression intensity was found only be associated with sex (*r* = 0.292, *p* = .037) due to moderate and strong positivity were only found in female patients. The percentages of positively stained glomerular cells with mTOR was significantly associated with tubulointerstitial area mTOR staining intensity (*r* = 0.444, *p* = .001). Tubulointerstitial area mTOR staining intensity was positively correlated with the PTEN expression (*r* = 0.331, *p* = .023) and negatively correlated with the degree of fibrosis in tubulointerstitial area (*r* = −0.288, *p* = .045). The percentages of stained tubulointerstitial area cells with mTOR was associated with the degree of fibrosis in tubulointerstitial area (*r* = −0.295, *p* = .038), the presence of crescent (*r* = 0.325, *p* = .021), and the degree of sclerosis in glomeruli (*r* = −0.297, *p* = .036). TGF-β1 expression is only associated with the one year survival of patients with pauci-immune glomerulonephritis (*r* = −0.291, *p* = .036). None of the samples of the patients died within the first year of diagnosis has more than 25% percentage staining with TGF-β1. However, baseline or post-treatment serum creatinine level or response to treatment was not found to be associated with mTOR pathway expression.

## Discussion

The mTOR is an intracellular serine/threonine kinase and one of the central components of a complex signaling pathway which is highly conserved in evolutionarily. mTOR is expressed ubiquitously in the cells of the body [6]. To study the mTOR activity in pauci-immune glomerulonephritis, we evaluated the expression of mTOR, PTEN and TGF-β1 in both glomerular and tubulointerstitial region of the kidney biopsy samples. The results of our study showed that mTOR pathway and it is regulators might contribute to the pathology seen in pauci-immune glomerulonephritis.

Recent studies revealed that mTORC1 might be one of the key regulators of podocyte size control which can be a compensation for podocyte losses due to podocyte injury [21]. It was also shown that mTORC2 may also play critical role for podocyte survival via ACT [22,23]. These findings suggest that mild mTOR activity might be a part of renal homeostasis [5]. There are also some evidences that abnormal mTOR activity may lead to renal lesions. In diabetic nephropathy, substantial mTORC1 activation was detected at early stages leading to an unbalanced cell hypertrophy, de-differentiation, podocyte detachment, and progressive glomerulosclerosis [24]. It was shown that in lupus nephritis model glomerular expression of mTOR and AKT were increased and they were observed mainly in the mesangium and the capillary loops due to increased expression by both podocytes and endothelial cells [25]. However little is known about the role of glomerular mTOR activity in crescentic glomerulonephritis. The only study evaluated 14 human kidney biopsies including anti-glomerular basement membrane glomerulonephritis, Henoch-Schonlein purpura nephritis, and anti-neutrophil cytoplasm autoantibody-associated glomerulonephritis [26]. The authors used S6K1 and eIF4E to get information about the activation mTORC1 signaling and showed substantially elevated staining in crescents and tubules from subjects with crescents in comparison weak staining in normal human kidneys. Our results indicate that a considerable amount of glomeruli and even more cells in tubulointerstitial area of patients with pauci-immune glomerulonephritis expressed mTOR. And mTOR expression in particular tubulointerstitial area might be related with the crescentic or sclerotic changes observed in glomeruli and fibrosis in interstitial area.

Our study also revealed that mTOR expression in tubulointerstitial area might also be operative in the pathology of pauci-immune glomerulonephritis. Recent evidence suggests that mTOR pathway could also be involved in normal tubular function. The inhibition of mTORC1 by sirolimus caused hypokalemia most probably due to renal potassium loss [27]. Sirolimus usage was also found to be associated with hypophosphatemia and phosphaturia with unknown cause. The mTOR inhibition may also lead delayed repair of ischaemia/reperfusion injury after kidney transplantation [22]. Although it seems that mTOR is necessary for normal tubular function it may also be involved in several tubular disorders. Tuberous sclerosis, which is a monogenic disease and causing cystic lesions and angiomyolipomas in kidneys as well as other multisystemic abnormalities, mutations in TSC1 or TSC2 leads hyperactivation of mTOR and mTOR inhibitors might be the first effective treatment for these patients [22,28]. In this study, we showed that mTOR was expressed in all tubulointerstitial areas of kidney biopsy samples with pauci-immune glomerulonephritis and the percentages of cells that is stained by mTOR is higher in tubulointerstitial area than glomerular region (*p* * =* .002). This study also revealed that mTOR expression in tubulointerstitial area might be related with glomerular mTOR expression and PTEN staining and fibrosis observed in tubulointerstitial area of pauci-immune glomerulonephritis. Therefore, one can speculate that if mTOR inhibitors beneficial they are acting on the tubulointerstitial compartment of the kidney.

The absence of normal control biopsy samples is the main limitation of this study. It precludes us to compare expression level of mTOR pathway with normal kidney tissue. The other limitation of this study is that we did not evaluate the phosphorylation or activation status of mTOR or it is up- or down-stream substrates.

## Conclusion

Our study showed that glomerular or interstitial expression of PI3K/AKT/mTOR pathway may play a role in the pathogenesis of pauci-immune glomerulonephritis and mTORC1 inhibitors might provide a viable alternative for this disease.
